# Zero-reflection acoustic metamaterial with a negative refractive index

**DOI:** 10.1038/s41598-019-40184-7

**Published:** 2019-03-04

**Authors:** Choon Mahn Park, Sang Hun Lee

**Affiliations:** 1 Dong-A University, Department of Materials Physics, Busan, 49315 South Korea; 2 Sogang University, Department of Physics, Seoul, 04107 South Korea

## Abstract

Waves are reflected to varying degrees at the boundary between two different media. A reduction of this reflection is important for many materials, including acoustic metamaterials. Here, we theoretically develop a balanced acoustic metamaterial that exhibits zero reflection between the metamaterial and air over its entire refractive index range, regardless of the sign of the refractive index. The metamaterial is realized using unit cell structures containing membranes and open tubes, and the material performance is verified experimentally, demonstrating sound reflectance of less than 10% in the frequency region that satisfies the homogeneous medium condition. The zero-reflection characteristic of the acoustic balanced metamaterial can improve the performance of new acoustic devices such as the invisibility cloak and super-lens and can also contribute to basic research of acoustic metamaterials.

## Introduction

Acoustic waves, along with electromagnetic waves, are widely used in everyday life in applications ranging from human verbal communication to medicine, industry and the military. The development of acoustic metamaterials that can manipulate sound propagation is important and is currently an area of active research^1,2^. When a wave passes through a boundary between two materials with different physical properties, reflection inevitably occurs at the interface between these materials, and such reflections can be a troublesome factor that causes performance deterioration in devices that use these waves. Therefore, the reduction of reflections at boundaries is of great scientific and engineering interest.

A metamaterial is a material that consists of an artificial structure satisfying the homogeneous medium condition (where the length of the unit cell <*λ*/4)^3^. Because metamaterials are capable of demonstrating new wave phenomena that are not seen in nature, research related to these materials is being actively conducted worldwide. However, even in a metamaterial, it is still difficult to prevent reflections at boundaries with normal materials. For example, because a reduced cloaking condition that included impedance mismatch was used for the study of invisibility cloaking, imperfect cloaking that resulted in an unavoidable reflection at the cloak surface was obtained^4–6^. Therefore, the development of a method to effectively remove reflections would be highly useful for a variety of metamaterial applications, such as the development of stealth functions. In addition, because resonance-based lumped elements are used to construct metamaterials in most cases^7–12^, the physical properties of the resulting metamaterials are strongly dispersive. In many cases, dispersive materials have disadvantages for use in specific applications; therefore, it is important to realize a non-dispersive metamaterial for practical applications.

The realization of the principle of an acoustic metamaterial that does not cause reflections at boundaries in all frequency regions while exhibiting positive and negative refractive index properties will be of major significance and will represent a new milestone in both academic and practical terms. To date, various methods have been proposed to solve the reflection problem in acoustics, including the use of the gradient index technique^13–15^, symmetry-breaking metamaterials^16^, and impedance matching of two media, in a specific refractive index range or at a specific angle of incidence^7,17–19^. However, in these cases, an anti-reflection property was only achieved at a specific refractive index range or angle of incidence, and there have been no reports to date of an anti-reflection property being achieved for all refractive indices, including positive and negative indices, and regardless of the angle of incidence.

However, in transmission line metamaterials, it has been established that the impedance of the metamaterial can be matched with that of the air when the balanced condition is satisfied^3^. This condition can be achieved by ensuring that the product of the shunt inductance and the capacitance has the same value as the product of the series inductance and the capacitance. In such a balanced metamaterial, reflections can be strongly suppressed and the transmission can be maximized over the entire refractive index range. Various applications of these metamaterials have been reported^20–22^. In this paper, based on membrane/open-tube composite structures^23^, we theoretically propose a balanced acoustic metamaterial that is impedance matched with air for all refractive indices including negative indices and verify the proposed metamaterial experimentally.

## Results

The constituent parameters that determine the propagation characteristics of acoustic waves in a material are the density of the medium $\rho$ and its bulk modulus *B*. The velocity of an acoustic wave in the medium *v*_*p*_ and the refractive index relative to air *n* are given by

1 $${v}_{p}=\sqrt{\frac{B}{\rho }},$$

2 $$n=\sqrt{\frac{{\rho }_{r}}{{B}_{r}}},$$

where ${B}_{r}=B/{B}_{0}$ and ${\rho }_{r}=\rho /{\rho }_{0}$ are the relative values of the bulk modulus and the mass density of the medium, respectively, with respect to the values in air, which are ${B}_{0}=1.42\times {10}^{5}\,{\rm{Pa}}$ and ${\rho }_{0}=1.22\,{\mathrm{kg}/m}^{3}$. The characteristic impedance of the medium for an acoustic wave *Z*_*m*,*c*_ is given by

3 $${Z}_{m,c}=\rho {v}_{p}=\sqrt{\rho B}.$$

When air and the medium are in contact, then the reflection coefficient (or reflectivity) *r* of the acoustic wave at the boundary is given by

4 $$r=\frac{{p}^{-}}{{p}^{+}}=\frac{{Z}_{m,c}-{Z}_{air,c}}{{Z}_{m,c}+{Z}_{air,c}},$$

where *Z*_*air*,*c*_, *p*^+^, and *p*^−^ are the characteristic impedance of the air and the pressure amplitudes of the incident and reflected waves, respectively^24^.

When open tubes (OTs) are installed periodically as lumped elements in a one-dimensional acoustic waveguide, the pressure amplitude in the waveguide is affected by the dynamic motion of the air column that exists in the OT, and the value of the bulk modulus thus changes^25^. In this case, the bulk modulus of the medium *B* is given by

5 $$B=\frac{{B}_{0}}{1-\frac{{\omega }_{{OT}^{2}}}{{\omega }^{2}}},$$

where the transition frequency of the bulk modulus is given by ${\omega }_{OT}=c\sqrt{S/l^{\prime} dA}$, and, if only OTs have been installed, the mass density of the metamaterial *ρ* is equal to that of air *ρ*_0_. Here, *c*, *S*, *l*′, *d*, and *A* are the speed of sound in air, the cross-sectional area of the OT, the effective length of the OT, the unit cell length, and the cross-sectional area of the waveguide, respectively.

When membranes of mass *M* are installed periodically as lumped elements within a one-dimensional acoustic waveguide, a dynamic restoring force is caused by the motion of the membrane. As a result, the motion of the medium is affected by this membrane motion (with acceleration), and the mass density of the medium *ρ* changes. If only thin membranes are installed in the waveguide, then the effective mass density of the metamaterial is

6 $$\rho =\rho ^{\prime} (1-\frac{{{\omega }_{mem}}^{2}}{{\omega }^{2}}),$$

while the bulk modulus of the medium is equal to that of air. Here, ${\omega }_{mem}=\sqrt{{k}_{mem}/\rho ^{\prime} Ad}$, $\rho ^{\prime} ={\rho }_{0}+M/Ad$, and *k*_*mem*_ are the transition frequency of the effective mass density, the static value of the effective mass density of the medium increased by the mass of the installed membrane, and the effective spring constant of the membrane, respectively^26^.

In the case of an acoustic metamaterial with a composite structure in which OTs and membranes are arranged alternately, each lumped element affects the constituent parameters of the medium independently. The static density of the medium then becomes *ρ*′ rather than *ρ*_0_ because of the effect of the membrane mass, and the transition frequency of the bulk modulus should be modified to take the form ${\omega }_{OT}=c\sqrt{\rho ^{\prime} S/{\rho }_{0}l^{\prime} dA}$, which comes from the continuity equation of the medium. As a result, the effective refractive index of the medium, *n*, is given by

7 $$n=\sqrt{\frac{\rho ^{\prime} }{{\rho }_{0}}}\sqrt{1-\frac{{{\omega }_{mem}}^{2}}{{\omega }^{2}}}\sqrt{1-\frac{{\omega }_{{OT}^{2}}}{{\omega }^{2}}}.$$

The refractive index can be negative or positive and can have imaginary values as the angular frequency *ω* varies.

In addition, the acoustic impedance of a one-dimensional composite acoustic metamaterial with an OT and a membrane *Z*_*m*_ is given by

8 $${Z}_{m}=\frac{{Z}_{m,c}}{A}=\sqrt{\frac{{\rho }_{0}l^{\prime} /S}{{A}^{2}/{k}_{mem}}}\sqrt{\frac{{(\omega /{\omega }_{mem})}^{2}-1}{{(\omega /{\omega }_{OT})}^{2}-1}}.$$

**Figure 1:**
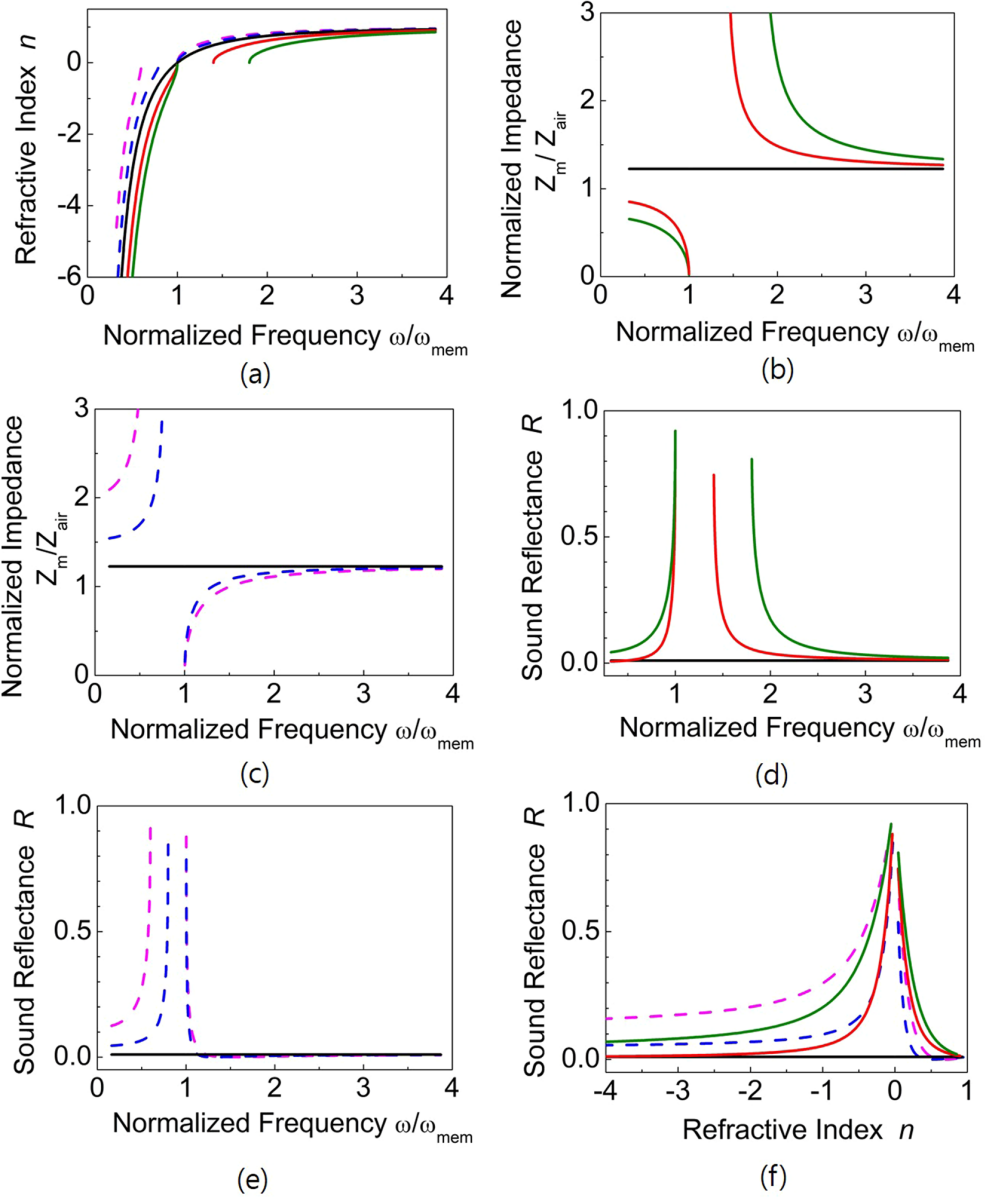
Physical properties of the acoustic metamaterial with a composite structure containing OTs and membranes. The magenta (dotted), blue (dotted), black (solid), red (solid), and green (solid) lines represent *ω*_*OT*_/*ω*_*mem*_ = 0.6, 0.8, 1.0, 1.4, and 1.8, respectively. (**a**) The refractive index of the composite acoustic metamaterial with normalized frequency. No forbidden band occurs when *ω*_*OT*_ = *ω*_*mem*_. (**b**,**c**) Normalized acoustic impedances of the composite metamaterials with respect to normalized frequency. The acoustic impedance is non-dispersive when *ω*_*OT*_ = *ω*_*mem*_. (**d**,**e**) Sound reflectance at the air/metamaterial boundary with respect to normalized frequency. The acoustic power reflection is approximately 1.2% when *ω*_*OT*_ = *ω*_*mem*_. (**f**) Change in reflectance with respect to refractive index.

If ${k}_{mem}={c}^{2}\rho {^{\prime} }^{2}S/{\rho }_{0}l^{\prime}$ and thus ${\omega }_{OT}={\omega }_{mem}$ (i.e., the balanced condition), then the imaginary term disappears from Eq. (7) and the acoustic impedance becomes a constant value (${Z}_{m}=\rho ^{\prime} c/A$) that is independent of frequency. This balanced condition can be achieved easily by varying the effective spring constant of the membrane *k*_*mem*_ and/or the effective length of the OT *l*′. If the balanced condition is satisfied and the membrane mass is very small, then ${Z}_{m}\simeq {Z}_{air}$ (where *Z*_*air*_ is the acoustic impedance of air). Therefore, no reflection occurs at the metamaterial/air boundary, irrespective of the sign of the refractive index of the metamaterial as its operating frequency changes. Figure 1 shows the change in the refractive index *n*, the normalized acoustic impedance *Z*_*m*_/*Z*_*air*_ and the sound reflectance of the metamaterial *R* (=|*r*|^2^) with $\rho ^{\prime} =1.22{\rho }_{0}$ as ${\omega }_{OT}$ and ${\omega }_{mem}$ are varied. As shown in Fig. 1, the balanced metamaterial marked with the black line does not have a forbidden band gap, and the reflectance is non-dispersive over the entire refractive index range. However, a forbidden band gap would exist in an unbalanced metamaterial. This is because in the bulk modulus and mass density, only one value is negative. In the case that ${\omega }_{OT}$ is 1.4 or 1.8 ${\omega }_{mem}$, the value of the bulk modulus is negative, and in the case that ${\omega }_{OT}$ is 0.6 or 0.8 ${\omega }_{mem}$, the value of the mass density is negative in the given frequency range. For a balanced metamaterial with $\rho ^{\prime} =1.22{\rho }_{0}$ and ${\omega }_{OT}={\omega }_{mem}$, calculation reveals that ${Z}_{m}=1.22{Z}_{air}$, and the sound reflectance *R* is approximately 1.02%.

**Figure 2:**
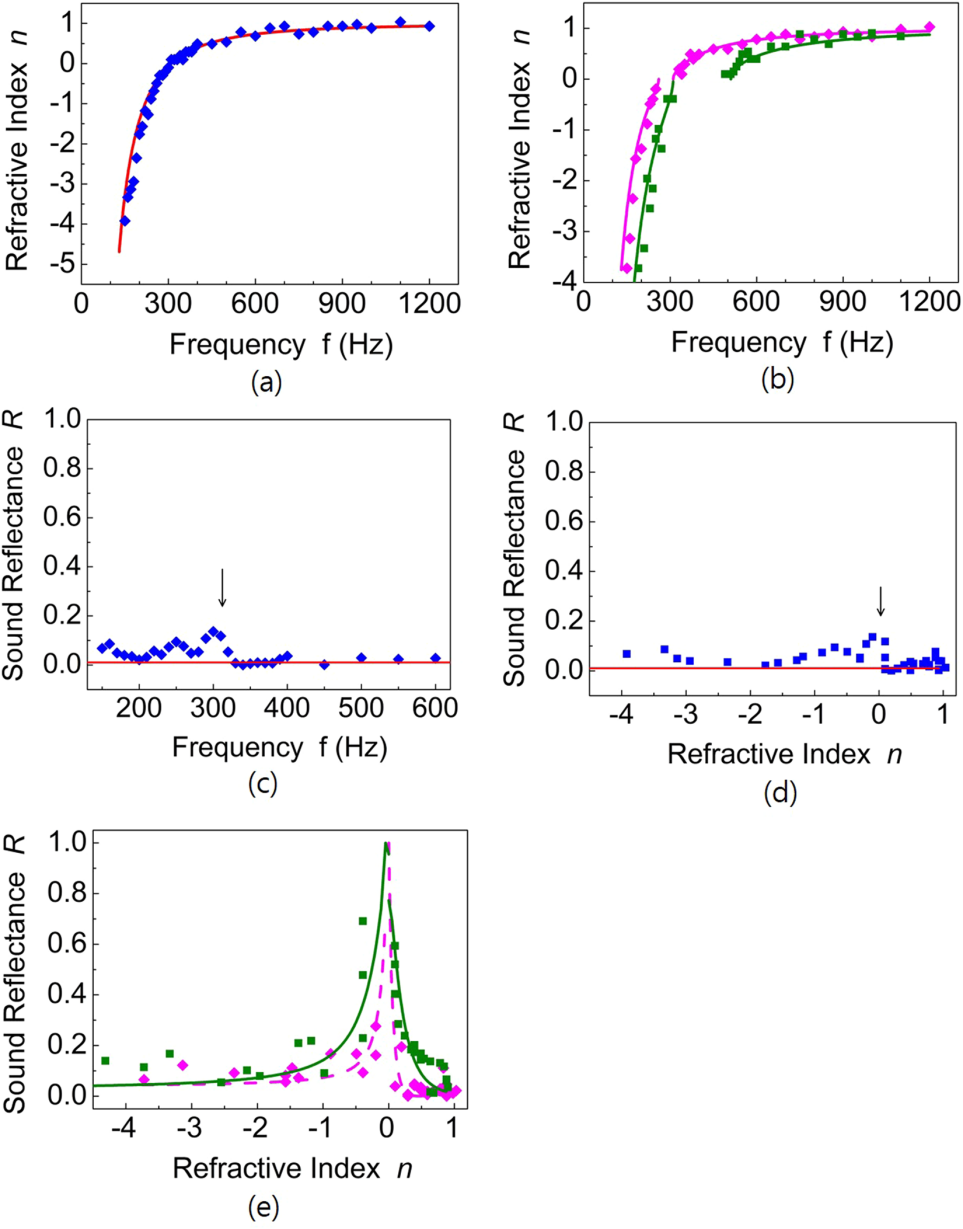
Experimental results. The marked points and corresponding lines indicate the results of measurements and calculations from theory, respectively. The arrow marks indicate singular points. (**a**) Refractive index vs. frequency when the metamaterial system is balanced; *ω*_*OT*_/*ω*_*mem*_ = 1.0. (**b**) Refractive index vs. frequency when the system is unbalanced; *ω*_*OT*_/*ω*_*mem*_ = 0.84 (magenta), *ω*_*OT*_/*ω*_*mem*_ = 1.64 (green). (**c**) Sound reflectance vs. frequency (balanced). (**d**) Sound reflectance vs. refractive index (balanced). (**e**) Sound reflectance vs. refractive index (unbalanced); *ω*_*OT*_/*ω*_*mem*_ = 0.84 (magenta), *ω*_*OT*_/*ω*_*mem*_ = 1.64 (green).

The measured results for the balanced acoustic metamaterial realized in experiments are shown in Fig. 2. Figure 2(a) shows the refractive index of the balanced acoustic metamaterial as a function of frequency. As shown in the figure, the experimental results are in good agreement with the theoretical results, and there is no forbidden band gap. In Fig. 2(b), we show the change in the refractive index *n* with frequency when ${\omega }_{OT}=\mathrm{\ 0.84}\,{\omega }_{mem}$ and ${\omega }_{OT}=1.64\,{\omega }_{mem}$ as typical examples of unbalanced acoustic metamaterials. As shown in the figure, if ${\omega }_{OT}\ne {\omega }_{mem}$, then a forbidden band gap occurs between ${\omega }_{OT}$ and ${\omega }_{mem}$.

Figure 2(c,d) show the changes in reflectance that occurred when ${f}_{mem}\simeq {f}_{OT}\simeq 308\,{\rm{Hz}}$ in the experiments. It is believed that the wiggles shown at 160 Hz and 250 Hz in Fig. 2(c) occur because of constructive interference in the metamaterial due to the three-medium structure in the experiment. Similar phenomena were observed at 650 and 1000 Hz, where the refractive index is positive (the reflectance data between 650 and 1200 Hz are not shown in Fig. 2(c), but are shown in Fig. 2(d), in which these data occur near $n=1$). While the reflections in the system are more strongly enhanced than those in a two-medium structured system at these frequencies, the measured sound reflectance value is still very small at less than 0.1. The increase in the reflectance observed near the frequency of 310 Hz occurs because ${\omega }_{mem}$ and ${\omega }_{OT}$ do not coincide exactly. If ${\omega }_{mem}$ and ${\omega }_{OT}$ have similar values but are not exactly matched, then the effect of a singular reflectance point appears at ${\omega }_{mem}$ $({\omega }_{OT})$, and *R* = 1 at this point. In Fig. 2(c), this singular point is indicated by a black arrow. In the experiments, ${\omega }_{mem}$ and ${\omega }_{OT}$ cannot be precisely matched with each other because of the effects of the membrane tension distribution and the effective length of the OT. Therefore, the reflectance increases near the frequency of 310 Hz. In this case, however, the reflectance remains close to the value of 0.1. The measured sound reflectance characteristics for typical unbalanced metamaterials with ${\omega }_{OT}=0.84\,{\omega }_{mem}$ and ${\omega }_{OT}=1.64\,{\omega }_{mem}$ are shown in Fig. 2(e). As shown in the figure, the sound reflectance is highly dispersive, with a large value in the −$0.5 < n < 0.5$ region.

## Discussion

We have presented a theoretical basis for a balanced acoustic metamaterial that not only has positive and negative refractive indices but also zero reflections from interfaces with normal materials. Using membranes and OTs, we realized a balanced acoustic metamaterial. We then measured the physical properties of a fabricated balanced acoustic metamaterial in the frequency region in which the homogeneous medium condition is satisfied. It was observed that the balanced metamaterial has either a positive or negative refractive index with no forbidden gap. While the reflectance was enhanced at some specific frequencies because the measurement system consists of three media, the resulting reflectance values were measured to be less than 10% within the measured refraction area. Because acoustic waves are widely used in medical, industrial and military applications, the range of fields in which this acoustic metamaterial can be applied is also very wide. The realization of an anti-reflection property using the proposed balanced acoustic metamaterial is very important for both medical and industrial purposes and will therefore make a significant contribution to the academic advancement of metamaterials.

## Methods

**Figure 3:**
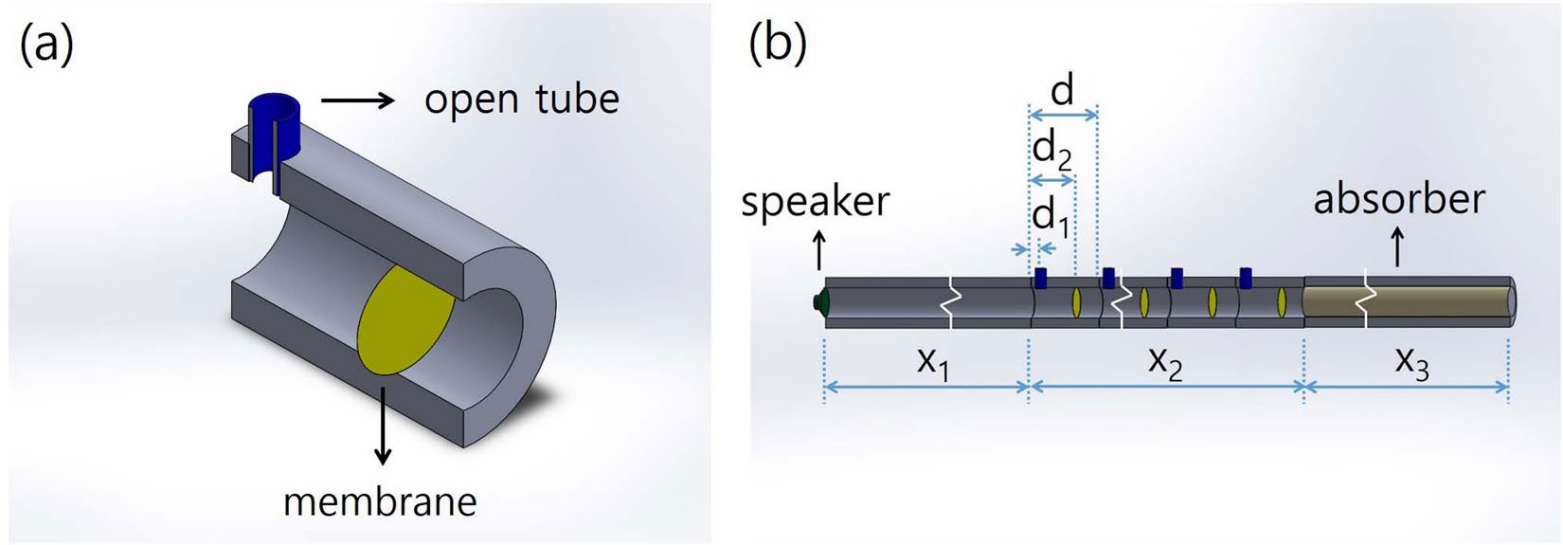
Schematic diagrams of the acoustic metamaterial and experimental setup. (**a**) Cross-sectional diagram of the unit cell. The inner diameters of the open tube and waveguide are 10 and 30 mm, respectively. The effective length of the open tube is varied from 11 to 45 mm. (**b**) Experimental setup. *d*_1_(=20 mm) and *d*_2_(=55 mm) indicate the distances of the open tube and membrane from the left side of the unit cell whose length is *d*(=70 mm). *X*_1_(=2.0 m), *X*_2_(=1.47 m) and *X*_3_(=50.0 m) indicate the lengths of the normal waveguide, metamaterial and absorber, respectively.

Figure 3(a) shows a schematic diagram of the unit cell of the composite metamaterial with the OT and membrane installed periodically to realize a balanced one-dimensional acoustic metamaterial. The unit cell made from a 10-mm-thick acrylic cylinder has a length of $d=70\,{\rm{mm}}$ and an inner diameter of 30 mm $(A=707\,{{\rm{mm}}}^{2})$. The OT made from a 2-mm-thick acrylic cylinder has an inner diameter of 10 mm. The OT and membrane are installed 20 mm and 55 mm away from the left side of the unit cell, respectively. The effective spring constant of the membrane, *k*_*m*_, is determined by applying uniform tension to a commercial polyvinyl chloride (PVC) thin film with a thickness of 0.013 mm. The static value of the effective mass density of the metamaterial, *ρ*′, is given by $\rho ^{\prime} \simeq 1.50\,{\mathrm{kg}/m}^{3}\simeq 1.23{\rho }_{0}$, where the membrane’s mass density is approximately $1.467\times {10}^{3}\,{\mathrm{kg}/m}^{3}$. Both *f*_*mem*_ (=*ω*_*mem*_/2*π*) and *f*_*OT*_ (=*ω*_*OT*_/2*π*) are determined by measuring the transition points of the normalized amplitudes of the acoustic waves in the metamaterial^27,28^. *f*_*mem*_ is determined to be 308 Hz. The value of *f*_*OT*_ can be varied between 260 and 510 Hz by varying the effective length of the OT *l*′ which is varied from 11 to 45 mm in the experiment.

The setup for the experiments is shown in Fig. 3(b). Sound waves generated by a speaker installed at the left end of the normal waveguide propagate through the normal waveguide and enter the metamaterial. The inner diameter and length of the normal waveguide are 30 mm and 2.0 m, respectively, and the corresponding values for the metamaterial are 30 mm and 1.47 m. The sound waves that pass through the metamaterial propagate to a long absorber with a diameter of 30 mm and length of 50 m. To simplify the consideration of the acoustic characteristics during the experiments, we ensured that the inner diameters of the normal waveguide, metamaterial, and absorber were all the same. The absorber located at the right end of the metamaterial absorbs acoustic energy, which results in negligibly small reflections; thus, the system behaves as if it extends to infinity. Therefore, our system can be regarded as a three-medium system consisting of an air-metamaterial-absorber combination. The pressure amplitude is measured using a miniature condenser-type microphone in both the incident waveguide and the metamaterial as a function of time and position. The reflection that occurs at the boundary between the metamaterial and the normal material is determined by measuring the standing wave ratio (SWR) of the acoustic wave along the left normal waveguide in the frequency range between 150 and 1200 Hz. The homogeneous medium condition is satisfied between these frequencies.

## References

[1] Ma G, Sheng P (2016). Acoustic metamaterials: From local resonances to broad horizons. Sci. Adv..

[2] Cummer SA, Christensen J, Alu A (2016). Controlling sound with acoustic metamaterials. Nature Reviews Materials.

[3] Caloz, C. & Itoh, T. *Electromagnetic Metamaterials*: *Transmission line theory and microwave applications.* (John Wiley & Sons, New Jersey, 2006).

[4] Schurig D (2006). Metamaterial electromagnetic cloak at microwave frequencies. Science.

[5] Yang Y, Wang H, Yu F, Xu Z, Chen H (2016). A metasurface carpet cloak for electromagnetic, acoustic and water waves. Sci. Rep..

[6] Zigoneanu L, Popa B-I, Cummer SA (2014). Three-dimensional broadband omnidirectional acoustic ground cloak. Nature Mater..

[7] Ma G, Yang M, Xiao S, Yang Z, Sheng P (2014). Acoustic metasurface with hybrid resonances. Nature Mater..

[8] Shelby RA, Smith DR, Schultz S (2001). Experimental verification of a negative index of refraction. Science.

[9] Fang N (2006). Ultrasonic metamaterials with negative modulus. Nature Mater..

[10] Wu Y, Lai Y, Zhang Z-Q (2011). Elastic metamaterials with simultaneously negative effective shear modulus and mass density. Phys. Rev. Lett..

[11] Brunet T (2015). Soft 3D acoustic metamaterial with negative index. Nature Mater..

[12] Naify CJ, Chang C-M, McKnight G, Nutt S (2010). Transmission loss and dynamic response of membrane-type locally resonant acoustic metamaterials. J. Appl. Phys..

[13] Park CM, Kim CH, Park HT, Lee SH (2016). Acoustic gradient-index lens using orifice-type metamaterial unit cells. Appl. Phys. Lett..

[14] Park CM, Lee SH (2018). Acoustic Luneburg lens using orifice-type metamaterial unit cells. Appl. Phys. Lett..

[15] Zhu R (2017). Bifunctional acoustic metamaterial lens designed with coordinate transformation. Appl. Phys. Lett..

[16] Dai H, Liu T, Xia B, Yu D (2017). Quasilossless acoustic transmission in an arbitrary pathway of a network. Phys. Rev. B.

[17] D’Aguanno G (2012). Broadband metamaterial for nonresonant matching of acoustic waves. Sci. Rep..

[18] Torrent D, Sanchez-Dehesa J (2007). Acoustic metamaterials for new two-dimensional sonic devices. New J. of Phys..

[19] Xie Y, Konneker A, Popa B-I, Cummer SA (2013). Tapered labyrinthine acoustic metamaterials for broadband impedance matching. Appl. Phys. Lett..

[20] Marteau A (2009). Ferroelectric tunable balanced right-and left-handed transmission lines. Appl. Phys. Lett..

[21] Borja AL, Belenguer A, Cascon J, Esteban H, Boria VE (2011). Wideband passband transmission line based on metamaterial-inspired CPW balanced cells. IEEE Antennas and Wireless Propag. Lett..

[22] Serrano E (2016). SiGe BiCMOS balanced transmission line based on coplanar waveguide and split ring resonator. Radio Science.

[23] Lee SH, Park CM, Seo YM, Wang ZG, Kim CK (2010). Composite acoustic medium with simultaneously negative density and modulus. Phys. Rev. Lett..

[24] Blackstock DT (2000). Fundamentals of Physical Acoustics.

[25] Bongard F, Lissek H, Mosig JR (2010). Acoustic transmission line metamaterial with negative/zero/positive refractive index. Phys. Rev. B.

[26] Lee SH, Wright OB (2016). Origin of negative density and modulus in acoustic metamaterials. Phys. Rev. B.

[27] Lee SH, Park CM, Seo YM, Wang ZG, Kim CK (2009). Acoustic metamaterial with negative density. Phys. Lett. A.

[28] Lee SH, Park CM, Seo YM, Wang ZG, Kim CK (2009). Acoustic metamaterial with negative modulus. J. of Phys.: Cond. Matter.

